# Rates and Correlates of Short Term Virologic Response among Treatment-Naïve HIV-Infected Children Initiating Antiretroviral Therapy in Ethiopia: A Multi-Center Prospective Cohort Study

**DOI:** 10.3390/pathogens8040161

**Published:** 2019-09-24

**Authors:** Birkneh Tilahun Tadesse, Adugna Chala, Jackson Mukonzo, Tolosssa Eticha Chaka, Sintayehu Tadesse, Eyasu Makonnen, Zabrina L. Brumme, Chanson J. Brumme, Eleni Aklillu

**Affiliations:** 1 Division of Clinical Pharmacology, Department of Laboratory Medicine, Karolinska Institute, Karolinska University Hospital Huddinge, 141 86 Stockholm, Sweden; eleni.aklillu@ki.se; 2 Department of Pediatrics, College of Medicine and Health Sciences, Hawassa University, Hawassa 1560, Ethiopia; 3 Department of Pharmacology, College of Health Sciences, Addis Ababa University, Addis Ababa 9086, Ethiopia; adugnadema@gmail.com (A.C.); ssintayehu2010@gmail.com (S.T.); eyasumakonnen@yahoo.com (E.M.); 4 Department of Pharmacology, College of Health Sciences, Makerere University, Kampala, Uganda; mukojack@yahoo.co.uk; 5 Adama General Hospital and Medical College, Adama 84, Ethiopia; tecb2006@gmail.com; 6 CDT Africa, College of Health Sciences, Addis Ababa University, Addis Ababa 9086, Ethiopia; 7 Faculty of Health Sciences, Simon Fraser University, Burnaby, BC V5A 1S6, Canada; zbrumme@cfenet.ubc.ca; 8 British Columbia Centre for Excellence in HIV/AIDS, Vancouver, BC V6Z 1Y6, Canada; cbrumme@cfenet.ubc.ca; 9 Department of Medicine, University of British Columbia, Vancouver, BC V5Z 1M9, Canada

**Keywords:** HIV, cART naïve, virologic outcome, pretreatment HIV drug resistance

## Abstract

There is limited data on virologic outcome and its correlates among HIV-infected children in resource-limited settings. We investigated rate and correlates of virologic outcome among treatment naïve HIV-infected Ethiopian children initiating cART, and were followed prospectively at baseline, 8, 12, 24 and 48 weeks using plasma viral load, clinical examination, laboratory tests and pretreatment HIV drug resistance (PDR) screening. Virologic outcome was assessed using two endpoints–virological suppression defined as having “undetectable” plasma viral load < 150 RNA copies/mL, and rebound defined as viral load ≥150 copies/mL after achieving suppression. Cox Proportional Hazards Regression was employed to assess correlates of outcome. At the end of follow up, virologic outcome was measured for 110 participants. Overall, 94(85.5%) achieved virological suppression, of which 36(38.3%) experienced virologic rebound. At 48 weeks, 9(8.2%) children developed WHO-defined virological treatment failure. Taking tenofovir-containing regimen (Hazard Ratio (HR) 3.1-[95% confidence interval (95%CI) 1.0–9.6], *p* = 0.049) and absence of pretreatment HIV drug resistance (HR 11.7-[95%CI 1.3–104.2], *p* = 0.028) were independently associated with earlier virologic suppression. In conclusion, PDR and cART regimen type correlate with rate of virologic suppression which was prominent during the first year of cART initiation. However, the impact of viral rebound in 38.3% of the children needs evaluation.

## 1. Introduction

The number of children accessing combination antiretroviral therapy (cART) has increased over the past decade following global initiatives including “test and treat” and the ambitious UNAIDS 90-90–90 treatment targets to help end the AIDS epidemic [1,2]. The 90-90-90 goals, which were originally hoped to be realized by 2020, aim to have 90% of people living with HIV being aware of their diagnosis; 90% of those with diagnosed infection be on sustained antiretroviral treatment, and 90% of those receiving antiretroviral treatment to have virological suppression [3]. Though these ambitious goals are critical in decreasing AIDS related morbidity and mortality in resource limited settings, recent data suggest that these targets may possibly be feasible in some settings [4], disparities in the ability of individual countries to perform towards the goals remain substantial [5,6]. Moreover, the global community currently fall very short of the targets: as of 2017, only 52% of children below the age of 14 years were receiving cART [1].

The main goal of long-term cART in HIV infected children is to achieve sustained virological suppression. Virological suppression is modulated by several factors including baseline viral load. In a European cohort of 420 HIV infected infants, virological suppression at 12 months of follow up was achieved in 89% of studied children, where lower baseline viral load and younger age at cART initiation were associated with faster virological suppression [7]. However, fewer data exist on the correlates of virologic suppression in resource-limited settings where pediatric antiretroviral treatment options are fewer [8,9]. Baseline viral load ≥ 100,000 copies per mL and delayed virological suppression were also associated with subsequent risk of virological rebound in a large (N = 53,000) study of HIV-infected adults across 20 major HIV treatment centers in France [10], but fewer data exist on the correlates and rates of virologic rebound in resource-limited settings. More importantly, data on the prevalence and impact of pretreatment HIV drug resistance mutations on pediatric firstline antiretroviral treatment regimen efficacy are also scarce for resource-limited settings [11,12,13,14]. 

In Ethiopia, high rates of virological suppression have been reported among HIV infected adults [15,16,17,18] and children [16], which are similar to figures reported for the rest of sub Saharan Africa [19]. However, increasing trends of acquired HIV drug resistance and treatment failure have also been reported in Ethiopia, at least in adults [20,21]. Moreover, a study among HIV-infected Ethiopian adults showed that presence of baseline drug resistance mutations was associated with lower rates of virologic suppression at months six and 12 months of cART [22]. Though pretreatment HIV drug resistance can substantially influence treatment outcomes, drug resistance information is not used to guide pediatric HIV clinical care in Ethiopia. This is because, in Ethiopia, neither routine plasma viral load monitoring nor HIV drug resistance testing are readily available to guide individualized patient care. Empiric choice of cART is therefore the routine practice. Indeed, the prevalence and impact of HIV drug resistance on firstline antiretroviral treatment outcomes in Ethiopian children living with HIV has not previously been characterized. 

Optimizing pediatric HIV treatment is critical to reduce child mortality and morbidity in resource limited settings, and to ensure that, as a global community, we meet the ambitious UNAIDS and Sustainable Development Goals by 2030. Local data are also crucial to inform policies and programmes for fast-track implementation of pediatric HIV treatment guidelines in such settings. To address the relatively paucity of data in pediatric HIV treatment outcomes in high HIV-burden settings, in particular the impact of baseline factors including pretreatment HIV drug resistance mutations on virological suppression these settings, we report the results of a multicenter prospective cohort study which assessed virological outcomes and associated factors at 8, 12, 24 and 48 weeks of initiation of cART among newly diagnosed HIV infected children attending HIV/AIDS clinics in Ethiopia. 

## 2. Methods 

### 2.1. Study Participants 

The Efavirenz Pediatric Dose Optimization Study (EPDOS) originally enrolled 111 cART naïve HIV-infected children at seven HIV/AIDS treatment centers across two of the largest administrative regions in Ethiopia: Oromia and Southern Nations Nationalities and Peoples Region (SNNPR), whose combined populations exceed 55 million [23]. The centers in Oromia region were: Adama General Hospital, Asela Referral and Teaching Hospital, and Shashemene General Hospital; from SNNPR: Hawassa University Comprehensive Specialized Hospital, Adare General Hospital, Otona Referral Hospital and Arbaminch General Hospital. These regions were selected because of their high HIV prevalence and the large and diverse population, which is generalizable to the whole country. In Ethiopia, the estimated HIV pediatric population is 62,000 (38,000–86,000) [24].

Participants were between the ages of 3–12 years, were cART naïve, whose parents/guardians consented to participate in the study and were able and willing to stay in follow up, and who had no acute severe illnesses at enrolment. Since Ethiopia practices the “test and treat” strategy, participants were enrolled within 2–4 weeks of HIV diagnosis after the required pretreatment counselling was completed. Children who had tuberculosis and those who had previously been on combination antiretroviral therapy were excluded, though PMTCT exposure was allowable. Note however that PMTCT history was not comprehensively available for study participants. Demographic, clinical and laboratory data were collected at baseline. Plasma viral load (pVL) was determined using the Real Time HIV-1 viral load test (Abbott, Des Plaines, IL, USA) using a 0.2 mL input volume of plasma.

### 2.2. Combination Antiretroviral Therapy

In accordance with the Ethiopian national guidelines for first line pediatric cART, all children were initiated on a regimen comprising two Nucleoside Reverse Transcriptase Inhibitors (lamivudine plus one of zidovudine, abacavir or tenofovir) (NRTIs) and one Non-nucleoside Reverse Transcriptase Inhibitor [2,25]. The NNRTI was efavirenz for all the children [25]. Prior to initiating cART, all participants received adherence counselling from professional nurse counsellors working in the HIV treatment center. The specific firstline cART regimen was selected by the clinician responsible for the care and follow up of the children. The study team did not have any role in the choice of cART regimen. 

### 2.3. Data Collection Procedures 

Clinical and laboratory measurements were collected at baseline (study enrollment) and at 8, 12, 24 and 48 weeks post-cART initiation. At baseline, a detailed clinical history, clinical examination and relevant laboratory tests were done. This included a detailed clinical assessment of symptoms, a physical examination, nutritional assessment (history and anthropometrics) and Karnofsky functional scoring. Baseline laboratory tests included plasma viral load (pVL), CD4 count, hemoglobin level, platelet count, and chemistry (aspartate aminotransferase (AST), alanine amino transferase (ALT), bilirubin, albumin, and blood urea nitrogen (BUN), Creatinine, and lipid profile levels). All clinical and laboratory tests performed at baseline were repeated at weeks 8, 12, 24 and 48 post-cART. Adherence counselling was also provided to all participants. Adherence was assessed using a composite of pill count, self-report and the visual analogue score (VAS) [26]—0% means they have taken none; 50% means they have taken half; and 100% means they have taken every single dose. Adherence of >95% was considered “optimal”.

### 2.4. HIV Drug Resistance Testing 

Baseline drug resistance testing was performed using dried blood spots (DBS) or dried plasma spots (DPS) as described in Tadesse et al. [27]. Briefly, using a standard ¼” manual hole punch or a pair of metal forceps, two spots (plasma or blood, as provided) per participant were transferred into sterile tubes for nucleic acid extraction [28]. The hole punch was cleaned of residual material between participant cards by punching 10 holes into clean filter paper [28]; the forceps were cleaned using bleach. Total nucleic acids were extracted using the NucliSENS easyMAG System according to manufacturer’s instructions (BioMerieux, Marcy-l’Étoile, France). 

HIV Protease and a portion of Reverse Transcriptase (RT) spanning a minimum of codons 1-234 were amplified via an initial Reverse Transcriptase step (Expand Reverse Transcriptase; Roche, Basel, Switzerland) followed by nested PCR (Expand Hifi System; Roche) or alternatively by RT-PCR (using the SuperScript III One-Step RT-PCR System with Platinum Taq High Fidelity DNA Polymerase; Invitrogen, MA, USA) followed by nested PCR (using the Expand HiFi System; Roche) [21]. Amplification was attempted using up to four oligonucleotide primer sets designed to amplify various HIV-1 group M subtypes: if amplification failed using the primary set, amplification was attempted using the backup sets. If amplification failed again, fresh nucleic acid extracts were prepared from remaining DPS or DBS and amplification was re-attempted as above. Amplicons were visualized on a 1% agarose gel and bulk (directly) sequenced on a 3130xl or 3730xl automated DNA sequencer (Applied Biosystems, Foster City, CA, USA). Chromatograms were analyzed using Sequencher version 5.0.1 (Gene Codes, Ann Arbor, MI, USA) or the automated basecalling software RECall [29], where nucleotide mixtures were called if the secondary peak exceeded 25% of the dominant peak *height* (Sequencher) or 17.5% of the dominant peak *area* (RECall).

However, in contrast to the previous manuscript which utilized the list of mutations for surveillance of transmitted HIV drug resistance defined by the World Health Organization (WHO) 2009 [30], drug resistance interpretations in the present study were performed using the Stanford HIVdb algorithm (version 8.8) [31]. This is because the present study explicitly assessed the impact of any pretreatment HIV drug resistance on individual-level virologic outcomes, whereas the main goal of the previous study was to characterize the burden of PDR in the pediatric setting, along with the specific mutations commonly observed, at the population level. Participants were classified as having PDR if resistance interpretation with Stanford HIVdb suggested low- to high-level resistance (score ≥15) to one or more ARVs

### 2.5. Virological Outcome Definitions

As the limit of detection of the Abbott Real Time HIV-1 viral load test is 150 copies/mL for the 0.2 mL starting volume of plasma used for analysis, a pVL < 150 copies HIV RNA/mL was considered “undetectable”. Virological rebound was defined as at least one pVL ≥ 150 copies/mL in a participant who had previously achieved a pVL of <150 copies/mL in a preceding follow up visit. Virological treatment failure (VTF) was defined according to the WHO guidelines of two consecutive pVL results ≥ 1000 copies per mL, recorded at least 3 months apart, after at least 6 months on cART [2] (that is, unlike the definition for virologic rebound, the definition of VTF did not require initial virologic suppression to <150 copies/mL).

### 2.6. Statistical Analysis

The relationships between baseline demographic, clinical and laboratory variables and achieving virologic outcomes of interest were assessed using univariable and multivariable and Cox proportional hazards regression. Kaplan-Meier survival analysis, with p-values computed using the log-rank test, were also undertaken for select baseline variables to graphically visualize relationships. The following variables were treated as binary or categorical variables, where the following reference categories were used. For sex, the reference category was “male”. Pretreatment drug resistance (PDR), tuberculosis (TB), isoniazid preventive therapy (IPT) and cotrimoxazole preventive therapy (CPT) were also treated as binary variables (“yes” vs. “no”), where “yes” was the reference category. Categorical variables included cART regimen (where abacavir-based regimen was considered the reference category) and WHO clinical staging (where clinical stage I was the reference category). Hazards for continuous variables were defined as follows: – age (per year decrement), AST and ALT (per unit/L decrement), Albumin, BUN, Creatinine and bilirubin (per mg/dL decrement), baseline viral load (per log_10_ copies/mL decrementCD4 count (per cells/uL increment), hematocrit (per 1% increment), total cholesterol, LDL, HDL, TG, WAZ, HAZ and BAZ (per mg/dL increment). In survival analyses, subjects who were lost to follow up or who died were censored at their last follow up timepoint. Covariates with a *p*-value of <0.2 in univariable analyses were included in the multivariable models. R software package (The R Foundation for Statistical Computing, Vienna, Austria) and GraphPad Prism version 7 (version 7, San Diego, CA, USA) were used for the analysis and presentation of the results. 

### 2.7. Ethics Statement

Ethical approval for this study was obtained from the Institutional Ethics Review Boards of Addis Ababa University, College of Health Sciences, SNNPR Regional Health Bureau, Karolinska Institutet in Stockholm, Simon Fraser University and Providence Health Care/University of British Columbia. The renewed National Research and Ethics Review Committee Ethics certificate is SHE/SM/14.3/0421/1/2019. Blood samples were collected after obtaining written informed consent in accordance with the tenets of the Declaration of Helsinki. For participants ≤12 years, written informed consent was obtained from their parent or guardian, while for participants >12 years of age, consent was obtained from the parent or guardian and assent obtained from the participant. All informed consent documents were provided in the local language.

## 3. Results

### 3.1. Baseline Characteristics of Study Participants 

**Figure 1:**
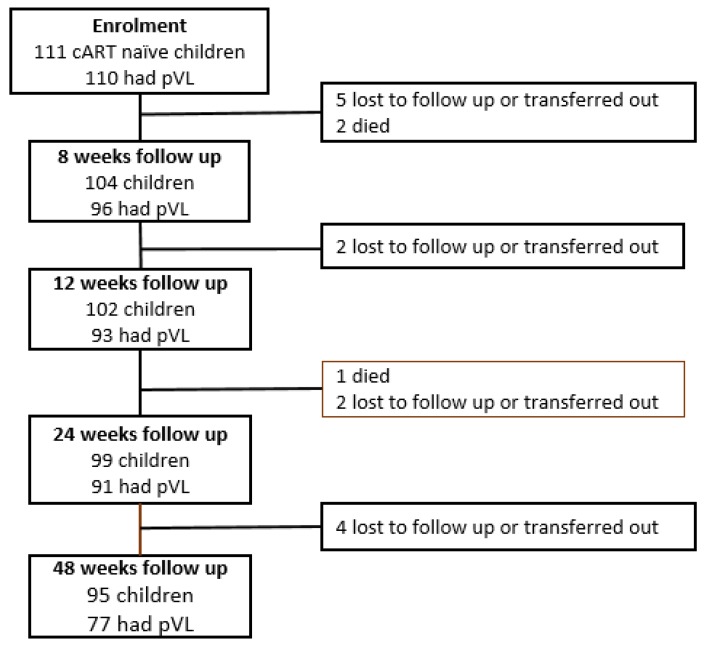
Enrolment and follow up during the first year of treatment. pVL = plasma viral load.

A total of 111 participants were recruited for study, for whom 110 (99.1%) had a baseline log_10_ pVL measurement (Figure 1). 

Additional baseline characteristics of cohort participants are detailed in Table 1. The median age of participants was 9 years (interquartile range: 5–12 years); the majority (56.8%) were male. The majority of the children, 44 (40%) were asymptomatic at cART initiation while 23 (20.9%), 33 (30%) and 10 (9.1%) were classified as being in WHO clinical stage 2, 3 and 4, respectively. Despite the relatively high prevalence of clinically-apparent symptoms however, participant functional Karnofsky scores (a self-reported measure of functional status that ranges from 0 to 100, where 100 indicates no health complaints), were high: 94 (82.5%) of participants recorded a Karnofsky functional score of 100 points at baseline. Most children, 59 (53.2%) were started on a tenofovir (TDF)/lamivudine (3TC)/efavirenz (EFV) regimen while 37 (33.3%) were initiated on an abacavir/3TC/EFV regimen and 15 (13.5%) were initiated on a zidovudine (AZT)/3TC/EFV regimen. Eighty-one (83.5%) and 34 (30.6%) were also initiated on co-trimoxazole preventive therapy (CPT) and isoniazid preventive therapy (IPT), respectively. The median (Q1–Q3) baseline CD4 count was 330 (178–741) cells/mL, and the median (Q1–Q3) log_10_ baseline pVL was 4.2 (3.3–4.9) copies/mL. Prior to initiating cART, all participants received optimal adherence counselling by a nurse counsellor. The level of adherence to treatment was reported as being optimal in all children during all follow up visits. 

Of the 51 participants for whom baseline resistance testing was successful, PDR was detected in six (11.8%). All participants with PDR had low- to high-level resistance to one or more ARVs. Specifically, five of the participants with PDR (83%) had intermediate- to high-level resistance efavirenz which may have compromised the efficacy of their initial cART regimen. The remaining participant with study-defined PDR had HIV that harbored the RT E138A mutation that confers low-level resistance to rilpivirine. No NRTI resistance was observed in any of the 51 participants for whom baseline resistance genotyping was successful. 

### 3.2. Rates and Correlates of Virological Suppression to Undetectable Levels

A total of 111 cART naïve HIV infected children were initially enrolled in the EPDOS cohort [23], but one participant did not participate in baseline viral load testing and subsequent follow-up (Figure 1). Follow up was therefore limited to the remaining 110 (99.1%) of participants. Over the study course, 13 children (11.8%) were either lost to follow up or were transferred out to another center out of the catchment region. An additional three children died: a 13 year boy died at home of unknown cause, and two girls, aged 6 and 10 years, who were initially classified as lost to follow up, were later confirmed to have died when the study team attempted to trace them (Figure 1). In total, 95 (86.4%) participants successfully completed all 48 weeks of follow up. 

Of the 110 HIV infected treatment naïve children who entered follow up, 94/110 (85.5%) achieved undetectable viremia, defined as pVL < 150 copies per mL, at some point during the study period. We explored clinical and laboratory correlates of virological suppression to pVL < 150, using Cox proportional Hazards regression. In univariable analyses, no baseline variables were statistically significantly associated with attaining pVL < 150 copies/mL during study follow-up (Table 2). However, a number of variables were weakly associated with attaining pVL < 150 copies/mL at p-values < 0.2. 

**Figure 2:**
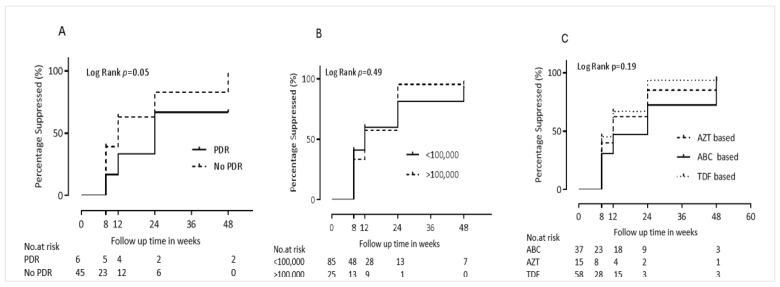
First year virological suppression (<150 copies per mL). Figure 2 represents the time to of virological suppression <150 copies per mL by pretreatment drug resistance status (**A**), baseline viral load (**B**) and NRTI drug type (**C**). PDR—Pre-Treatment HIV Drug resistance; AZT—Zidovudine; ABC—Abacavir; TDF—Tenofovir.

Specifically, the following baseline characteristics were identified as protective (that is, they were associated with a greater rate of achieving undetectable viremia in univariate analyses). Each decrease of one integer unit of body-mass-index-for-age Z-score (BAZ) was associated with a 10% increased rate of achieving undetectable viremia (Hazard Ratio (HR) 1.1 [95% CI 0.9–1.3], *p* = 0.09. Being on a TDF-containing cART regimen was associated with a 40% higher rate of achieving undetectable viremia (HR 1.4 [95% CI 0.9–2.1] compared to participants on other regimen types (*p* = 0.18). The relationship between virological suppression to undetectable levels and NRTI drug type contained in first line cART is also depicted using Kaplan-Meier curves in Figure 2. Moreover, lacking pre-treatment drug resistance (PDR) was associated with a 2.6-fold increased rate of achieving undetectable viremia (Hazard Ratio 2.6 [95% CI 0.8–8.6]) compared to participants with PDR (*p* = 0.12). The relationship between PDR and time to achieving pVL <150 copies/mL is also graphically illustrated in Figure 2. The following baseline characteristics were identified as “risk” (that is, they were associated with *decreased* rate of achieving undetectable viremia in univariate analyses). Not taking isoniazid preventive therapy (IPT) was associated with 30% decreased rate of achieving virological suppression (HR 0.7 [95% CI 0.5–1.2], *p* = 0.19). Similarly, being classified into WHO clinical stage 2 or stage 3 was associated with 30% decreased rate of achieving undetectable viremia (HR 0.8 - [95% CI 0.7–1.1]) compared to being classified into stage 1 (*p* = 0.08, data not shown). Each decrease in baseline viral load by one log_10_ copies per ml was associated with a 20% decreased rate of achieving undetectable viremia (HR 0.8 - [95% CI 0.7–1.1], *p* = 0.13). Virological suppression to undetectable levels stratified by baseline viral load is graphically illustrated using Kaplan-Meier curves in Figure 2. Each decrease in baseline low density lipoprotein (LDL) by one mg/dl was associated with a 10% shorter time to achieving undetectable viremia (HR 0.9 - [95% CI 0.9–1.0], *p* = 0.17). 

A multivariable model was constructed using all baseline parameters with *p*-values of < 0.2 in the univariable analyses, with the exception of body-mass-index-for-age Z-score (BAZ). The latter was excluded to avoid the possibility of collinearity, as both height-for-age Z-score (HAZ) and weight-for-age Z-score (WAZ) were included in the model, and BAZ is a function of weight and height. After adjustment for all baseline factors included in the model, the only variables that remained independently associated with time to virological suppression < 150 copies per mL were pretreatment drug resistance and type of cART regimen were (Table 2). Lacking pretreatment HIV drug resistance was associated with an 12 times higher rate of virologic suppression to undetectable levels compared to having pretreatment HIV drug resistance (HR: 11.1 95% CI: 1.3–94.7, *p*-value = 0.028). Being on a TDF based cART regimen was associated with 3.1 times shorter time to virological suppression compared to being on an ABC based regimen (HR: 3.1; 95% confidence interval – 95% CI: 1.0–9.6; *p*-value = 0.049).

### 3.3. Rates and Correlates of Subsequent Virologic Rebound

We next assessed rates of virologic rebound defined as registering at least one pVL ≥ 150 copies/mL after having first achieved a pVL of < 150 copies/mL; as such, this analysis was restricted to the 94 children who had achieved virological suppression at least once during follow up. Of these 94 children, 36 (38.3%) subsequently experienced virological rebound. Specifically, of these 36 children, 11 (30.6%), who were suppressed at week 8 had a rebound at week 12; another 11 children (30.6%) who had achieved suppression at weeks 8 and/or 12 had rebound at week 24; and, 14 (38.9%) children who had achieved one or more virological suppression at weeks 8, 12 and/or 24 had viral rebound at week 48. The median of the peak viral rebound was 2.2 (Q1–Q3: 2.2–3.0) log_10_ copies/mL.

**Figure 3:**
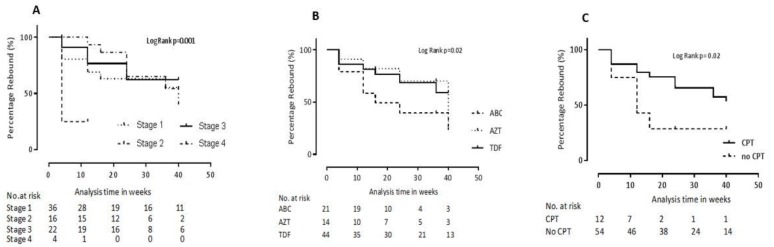
Virological rebound during the first year of antiretroviral therapy. Figure 3 represents virological rebound stratified by WHO clinical stage (**A**), cART regimen (**B**) and cotrimoxazole preventive therapy (CPT) status (**C**). Stage 1–4 represent WHO clinical staging; ABC–abacavir; AZT—zidovudine; TDF—tenofovir; No. at risk—Number at risk.

We next employed Cox proportional Hazards regression to characterize the relationship between participant clinical and demographic characteristics and risk of virologic rebound. Of note, the variables investigated in the present analysis were those measured at baseline, not at the time of initial virologic suppression. On univariable analysis, a decrease by one year of age was associated with a 10% lower rate of viral rebound (HR 0.9 [95% CI 0.8–1.0], *p* = 0.12). Similarly, taking a TDF-containing firstline regimen resulted in a 60% reduction in the hazard of virologcal rebound (HR 0.4 - [95% CI 0.2–0.9], *p* = 0.027). The trend of virological rebound stratified by the NRTI drug contained in firstline regimen is depicted using Kaplan-Meier curves (Figure 3). Not taking CPT was also associated with a 60% reduction in the hazard of virological rebound (HR 0.4 - [95% CI 0.2–0.9], *p* = 0.04). On the other hand, being WHO clinical stage 4 increased the hazard of virological suppression by 4-fold (HR 4.0 - [95% CI 1.1–14.3], *p* = 0.036). The relationship between CPT status and WHO clinical stage with the trend of virological rebound is described using Kaplan-Meier curves in Figure 3. Not taking IPT also increased the risk of virological rebound by 1.7-fold (HR 1.7 - [95% CI 0.8–3.6], *p* = 0.18) (Table 3).

A multivariable cox proportional hazards model was constructed using all the variables with p-value < 0.2. After controlling for confounders, all the covariates did not reach statistical significance expect WHO clinical stage which showed a weak association. Specifically, being classified as WHO clinical stage 4 disease resulted in a 3.6 times increased hazard of virologic rebound (HR 3.6 - [95% CI 0.7–18.8], *p* = 0.13) (Table 3).

Of note, of the 36 children who experienced viral rebound following initial suppression according to the above definition, 30 (83.3%) registered only a single pVL result ≥ 150 copies per mL after which they later re-achieved virological suppression to < 150 copies/mL. The remaining 6 children (16.7%) never re-achieved suppression, and fulfilled the WHO definition of virological treatment failure (VTF), defined as two consecutive pVL results ≥ 1000 copies per mL, recorded at least 3 months apart, after at least 6 months on cART [2]. Indeed, the end of the follow up period, 9 of the original 110 study participants (8.2%) had fulfilled the definition of VTF according to the WHO guidelines: these included the latter 6 participants as well as three additional children who never achieved pVL suppression < 150 copies/mL at any point during study follow up.

## 4. Discussion

Achieving sustained virologic suppression following initiation of cART is critical to ensure reduction in mortality and morbidity of HIV infected children, but limited data exist on the rates and correlates of virologic response among HIV-infected children in East and Southern Africa (ESA). Several factors could affect virological suppression among HIV infected children including effective cART regimens, optimal adherence to treatment and other clinical-laboratory predictors [32]. To our knowledge, this the first prospective study of cART naïve HIV infected children in Ethiopia that sought to evaluate the association between baseline demographic and clinical variables, including the presence of primary HIV drug resistance, with virologic outcomes in the first year of cART initiation. Moreover, in Ethiopia, there is no disaggregated national pediatric data on the rate of sustained virological suppression among HIV infected children who initiated cART (that is, the statistic represented by the third “90” of the UNAIDS 90-90-90 goal) [5]. Only an aggregate estimate of 86% virological suppression in 2016 has been reported, which is higher than the ESA regional average of 52% [33,34,35]. The current study revealed high initial rates of virological suppression - 85.5% of participants achieved pVL <150 copies/mL at some point during study follow up - a rate that is comparable to reports by other studies [8,9,17,18]. Our study further revealed that, after adjusting for other baseline factors, the presence of PDR and the use of abacavir-containing first-line cART regimen were associated with lower rates of virologic suppression.

Even though a high proportion of children initially achieved virological suppression, a substantial proportion (38.3%) subsequently registered at least one pVL ≥ 150 copies per mL, a rate that is similar to a study from South Africa [35]. Those who were classified as being WHO clinical stage 4 had an increased hazard of virologic rebound. Promisingly however, the majority (~80%) of participants who experienced viral rebound went on to re-achieve pVL suppression, suggesting that most were transient viral “blips” rather than an indication of virologic treatment failure (VTF). Indeed, VTF rates were relatively low: only nine of the original 110 study participants (8.2%) fulfilled the WHO definition of VTF at the end of the study period. Taken together, the findings show that newly diagnosed HIV infected children who are started on standard cART regimes, as recommended by the WHO guidelines [2], generally do well virologically at 48 weeks of treatment.

The impact of PDR on virologic outcomes among newly-diagnosed HIV infected children is not well established in resource-limited settings [22,36,37,38,39]. In theory, PDR could compromise the efficacy of first-line cART regimens, thereby reducing rates of virological suppression and increasing subsequent rates of virological treatment failure. However, previous studied have reported conflicting results. Several African and multinational studies reported a positive association between PDR and virologic suppression while other studies and in contrast to other African and Asian studies reported the lack of any association between PDR and virological suppression [39,40]. Our study showed that, after adjusting for other baseline factors, presence of any PDR was associated with reduced rates of virological suppression.

The difference in virological suppression between HIV infected children with PDR and those without is significant after the third month of cART indicating that more children with PDR continue to have high viral load. Interestingly our result indicates no significant association of PDR with risk of viral rebound. Despite several studies which reported the relevance of baseline viral load as a predictor of virologic outcome [10,41,42], our study demonstrated no association between the level of baseline viral load and virologic response.

Antiretroviral regimens types have differing safety and efficacy outcomes among HIV infected children. All the children who were included in the current cohort were started on an efavirenz based cART regimen based on the WHO 2016 consolidated guideline [2], which limited our ability to compare the impact of NNRTI drug types on virological outcomes. All children received lamivudine as one component of the NRTI backbone, therefore regimens only differed in terms of the second NRTI prescribed. A tenofovir based regimen was found to be associated with earlier virological suppression as compared to an abacavir based cART regimen, while no statistically significant difference was observed for zidovudine based regimens. However, studies have shown comparable antiviral activity between tenofovir/lamivudine and abacavir/lamivudine combined with dolutegravir and abacavir/lamivudine versus tenofovir/ emtricitabine [43,44,45]. The similar findings in previous reports could be because of the differing third agent in the cART combination and warrants more investigation.

Our study has several strengths including the prospective follow up ensuring higher data quality, and assessment of pretreatment HIV drug resistance. It also assessed a wide range of clinical and laboratory parameters to determine their association with virologic outcome. However, the small sample size in certain subgroups could have limited the statistical power of the study. Nearly 12% of the participants were transferred out or were lost to follow up further decreasing the sample size for the last follow up. The low success rate of HIV drug resistance genotyping might decrease the power to find any associations. However, despite this limitation, participants in whom genotyping was successful versus not did not differ with respect to their baseline characteristics except albumin levels. Moreover, anyone with PDR was treated as “resistant” regardless of whether the mutations conferred decreased susceptibility to the regimen they were on.

In conclusion, the study showed that a high proportion of HIV infected children achieve virological suppression at 48 weeks of cART. Pretreatment HIV drug resistance is associated with delayed virological suppression while a tenofovir based cART regimen is independently associated with earlier virological suppression during the first year of treatment. Our study also showed that there is a high prevalence of viral rebound, although these episodes were primarily transient virological “blips” and generally did not precede virological treatment failure. Our findings indicate the need for individualized patient care decisions based on presence of drug resistance mutations at baseline. Furthermore, it calls for more studies with larger sample size to understand the relationship between PDR, treatment regimen and virologic response in pediatric populations.

## Figures and Tables

**Table 1:** Baseline characteristics of participants in the cohort (2017–2019).

Variable	Frequency (%)	N with Data
Age at enrollment, median (Q1–Q3), years	9 (5–12)	111
Sex, n (%) Male	63 (56.8)	111
Any PDR, n (%) Yes	6 (11.8)	51
Tuberculosis, n (%) Yes	16 (16.4)	111
Isoniazid preventive therapy, n (%) Yes	34 (30.6)	111
Cotrimozazole preventive therapy, n (%) Yes	81 (83.5)	97
Hematocrit, median (Q1–Q3) in %	37.3 (35.1–40.5)	84
CD4+ T cell count, median (Q1–Q3)	330 (178–741)	59
Aspartate aminotransferase, median (Q1–Q3) units/L	38 (30–48)	108
Alanine aminotransferase, median (Q1–Q3), units/L	27 (20–38)	106
Total Bilirubin, median (Q1–Q3) mg/dL	0.8 (0.4–1.1)	105
Albumin, median (Q1–Q3) g/dL	3.8 (3.2–4.2)	104
Blood urea nitrogen, median (Q1–Q3) mg/dL	18 (12.1–26.5)	108
Creatinine, median (Q1–Q3) mg/dL	0.6 (0.4–0.7)	107
Total cholesterol, median (Q1–Q3) mg/dL	120 (96–150)	106
cART Regimen		111
Abacavir-based	37 (33.3)	
Zidovudine-based	15 (13.5)	
Tenofovir-based	59 (53.2)	
WHO clinical stage at enrolment		110
Stage 1	44 (40)	
Stage 2	23 (20.9)	
Stage 3	33 (30)	
Stage 4	10 (9.1)	
Low density lipoprotein, median (Q1–Q3) mg/dL	45 (32–63)	108
High density lipoprotein, median (Q1–Q3) mg/dL	47 (38–65)	105
Triglyceride, median (Q1–Q3) mg/dL	103 (87–151)	106
Weight for age Z score, median (Q1–Q3) mg/dL	–1.2 (–2.5–(–0.6))	73
Height for age Z score, median (Q1–Q3) mg/dL	–1.4 (–2.5–(–0.6))	109
Body mass index Z score, median (Q1–Q3) mg/dL	–1.2 (–2.4–(–0.3))	109
Log_10_ plasma viral load, median (Q1–Q3) copies/mL	4.2 (3.3–4.9)	110

Q1–Q3—Interquartile range; n—number; PDR—Pretreatment drug resistance.

**Table 2:** Baseline factors associated with achieving undetectable viremia (pVL < 150 copies per mL) during follow up: univariable and multivariable analysis.

Variable	Virological Suppression		Crude HR (95% CI)	*P* Value	aHR (95%CI)	*P* Value
	Undetectable	Detectable				
Age in years: median (Q1–Q3)	9 (5–12.5)	9 (5.5–12.5)	1.0 (0.9–1.1)	0.82		
Sex: N (%)						
Male	57 (60.6)	6 (37.5)	Ref	Ref		
Female	37 (39.4)	10 (62.5)	1.1 (0.7–1.6)	0.76		
PDR: N Yes (%)						
Yes	3 (7.3)	3 (30)	Ref	Ref	Ref	Ref
No	38 (92.7)	7 (70)	2.6 (0.8–8.3)	0.12	11.7 (1.3–104.2)	0.028
TB: N (%)						
Yes	13 (14)	3 (18.8)	Ref	Ref		
No	80 (86)	13 (81.2)	1.0 (0.5–1.8)	0.96		
IPT: N (%)						
Yes	29 (30.9)	4 (25)	Ref	Ref	Ref	Ref
No	65 (69.2)	12 (75)	0.7 (0.5–1.2)	0.19	0.6 (0.1–4.5)	0.64
CPT: N (%)						
Yes	65 (82.3)	12 (85.7)	Ref	Ref		
No	14 (17.7)	2 (14.3)	1.0 (0.5–1.7)	0.92		
HCT in %: median (Q1–Q3)	37.7 (35.1–41.2)	36.7 (35.5–39)	1.0 (1.0–1.1)	0.55		
CD4 cells/mm^3^ median (Q1–Q3)	381 (223–741)	217 (178–317)	1.0 (0.9–1.0)	0.95		
AST in units/dL: median (Q1–Q3)	37 (30–46)	45 (37–60)	1.0 (0.9–1.0)	0.31		
ALT in units/dL: median (Q1–Q3)	26 (18–36)	39 (33–42)	1.0 (0.9–1.0)	0.47		
Bil-T in mg/dL: median (Q1–Q3)	0.7 (0.4–1.1)	0.9 (0.5–1.1)	1.06 (0.8–1.4)	0.68		
ALB in mg/dL: median (Q1–Q3)	3.8 (3.2–4.3)	3.8 (3.5–4.1)	1.0 (0.8–1.3)	0.95		
BUN in mg/dL: median (Q1–Q3)	17 (12–24)	20.5 (15–36)	1.0 (0.9–1.0)	0.27		
Cr in mg/dL: median (Q1–Q3)	0.6 (0.4–0.7)	0.6 (0.4–0.7)	1.3 (0.6–2.7)	0.54		
TChol in mg/dL: median (Q1–Q3)	122 (98–152)	103 (65–142)	1.0 (0.9–1.0)	0.70		
cART Regimen, N (%)						
ABC	34 (91.9)	3 (8.1)	Ref	Ref	Ref	Ref
AZT	13 (86.7)	2 (13.3)	1.2 (0.6–2.3)	0.57	4.6 (0.1–25.8)	0.08
TDF	47 (81)	11 (19)	1.4 (0.9–2.1)	0.18	3.1 (1.0–9.6)	0.049
WHO Stage, N (%)						
1	38 (88.4)	5 (11.6)	Ref	Ref	Ref	Ref
2	21 (91.1)	2 (9.9)	0.7 (0.4–1.1)	0.14	0.6 (0.1–6.0)	0.64
3	29 (87.9)	4 (12.1)	0.7 (0.4–1.1)	0.09	1.0 (0.1–8.1)	0.99
4	5 (50)	5 (50)	0.6 (0.2–1.5)	0.28	1.1 (0.1–9.0)	0.93
LDL in mg/dL: median (Q1–Q3)	45 (32–63)	43 (35–56)	0.9 (0.9–1)	0.17	1.0 (0.9–1.0)	0.45
HDL in mg/dL: median (Q1–Q3)	47 (38–65)	45 (33–59)	0.9 (0.9–1)	0.42		
TG in mg/dL: median (Q1–Q3)	106 (89–157)	91 (69–144)	1.0 (0.9–1)	0.95		
WAZ in ZS: median (Q1–Q3)	−1.1 (−2.2– (−0.5))	−2.6 (−3.4– (−1.1))	1.0 (0.9–1.1)	0.11	1.0 (0.6–1.6)	0.92
HAZ in ZS: median (Q1–Q3)	−1.4 (−2.3– (−0.6))	−1.7 (−3.0– (−0.6))	1.0 (0.9–1.1)	0.13	7.4 (0.2–223)	0.25
BAZ in ZS: median (Q1–Q3)	−1.0 (−2.2– (−0.1))	−1.9 (−3.1– (−1.0))	1.1 (0.9–1.3)	0.09	**	**
Log_10_pVL: median (Q1–Q3)	4.2 (3.2–5.0)	4.3 (4.1–5.0)	0.8 (0.7–1.1)	0.13	1.1 (0.6–2.3)	0.69

PDR—pretreatment drug resistance; TB—Tuberculosis, all forms; IPT—isoniazid preventive therapy; CPT—co-trimoxazole preventive therapy; HCT—hematocrit; AST—aspartate transferase; ALT—alanine aminotransferase; Bil-T—total bilirubin; ALB –serum albumin; BUN—blood urea nitrogen; LDL—low density lipoprotein; TG—triglyceride; WAZ—weight-for-age Z score; BAZ—body mass index-for-age Z score; Log10 pVL—baseline viral load (after log transformation); cART—combination antiretroviral therapy; HR—Hazard ratio; aHR—adjusted hazard ratio. ** To avoid collinearity, BAZ (which is a function of weight and height) was excluded from the multivariate model. The following categories were treated as binary variables where “yes” is the reference group—PDR, TB, IPT and CPT. The reference groups for categorical variables are indicated on the table. The following were continuous variables, where hazards were defined in terms of unit decrement—age, AST, ALT, Albumin, BUN, Creatinine, bilirubin and baseline viral load. The following were continuous variables, where hazards were defined in terms of unit increment—CD4 count, hematocrit, total cholesterol, LDL, HDL, TG, WAZ, HAZ and BAZ.

**Table 3:** Predictors of virological rebound among cART naïve HIV infected children who achieved virological suppression while on first line antiretroviral therapy (n = 94).

Variable	Rebound		*P* Value	HR (95% CI)	aHR (95%CI)	*P* Value
	Yes	No				
Age in years: median (Q1–Q3)	7 (5–13)	9 (7–12)	0.12	0.9 (0.8–1.0)	0.9 (0.8–1.1)	0.45
Sex: N (%) Male Female	21 (58.3) 15 (41.7)	36 (62.1) 22 (37.9)	Ref 0.67	Ref 1.2 (0.6–2.2)		
PDR: N (%) Yes No	2 (18.2) 9 (81.8)	1 (3.3) 29 (96.7)	Ref 0.26	Ref 0.4 (0.1–1.9)		
TB: N (%) Yes No	32 (88.9) 4 (11.1)	48 (84.2) 9 (15.8)	Ref 0.93	Ref 1.0 (0.3–2.7)		
IPT: N (%) Yes No	9 (25) 27 (75)	20 (34.5) 38 (65.5)	Ref 0.18	Ref 1.7 (0.8–3.6)	Ref 2.2 (0.6–7.2)	Ref 0.22
CPT: N (%) Yes No	22(75.9) 7 (24.1)	43 (86) 7 (14.0)	Ref 0.04	Ref 0.4 (0.2–0.9)	Ref 1.1 (0.2–4.2)	Ref 0.94
HCT in %: median (Q1–Q3)	37.4 (32.9–41.2)	37.8 (35.9–40.9)	0.75	1.0 (0.9–1.1)		
CD4 cells/mm^3^ median (Q1–Q3)	447 (223–744)	330 (161–741)	0.85	1.0 (1.0–1.0)		
AST in units/dL: median (Q1–Q3)	37 (33-–54)	37 (29–45)	0.50	1.0 (1.0–1.0)		
ALT in units/dL: median (Q1–Q3)	27 (17–38)	25 (20–34)	0.37	1.0 (1.0–1.0)		
Bil-T in mg/dL: median (Q1–Q3)	0.8 (0.4–1)	0.6 (0.4–1.1)	0.78	0.9 (0.5–1.6)		
ALB in mg/dL: median (Q1–Q3)	3.8 (3–4.3)	3.8 (3.3–4.2)	0.83	1.0 (0.6–1.4)		
BUN in mg/dL: median (Q1–Q3)	18 (12–24)	15.3 (12–25)	0.51	1.0 (0.9–1.0)		
Cr in mg/dL: median (Q1–Q3)	0.5 (0.4–0.6)	0.6 (0.5–0.8)	0.36	0.6 (0.2–1.9)		
TChol in mg/dL: median (Q1–Q3)	108 (89–142)	128 (99–155)	0.09	1.0 (1.0–1.0)	1.0 (0.9–1.0)	0.33
cART Regimen, N (%) ABC AZT TDF	15 (41.7) 5 (13.9) 16 (44.4)	19 (32.8) 8 (13.8) 31 (53.5)	Ref 0.25 0.027	Ref 0.5 (0.2–1.5) 0.4 (0.2–0.9)	Ref 1.0 (0.2–4.2) 1.1 (0.3–4.1)	Ref 0.96 0.88
WHO Stage, N (%) 1 2 3 4	18 (51.4) 6 (17.1) 8 (22.7) 3 (8.6)	20 (34.5) 15 (25.9) 21 (36.2) 2 (3.5)	Ref 0.46 0.37 0.036	Ref 0.7 (0.3–1.8) 0.7 (0.3–1.6) 4.0 (1.1–14.3)	Ref 0.4 (0.1–1.6) 0.6 (0.2–1.8) 3.6 (0.7–18.8)	Ref 0.22 0.38 0.13
LDL in mg/dL: median (Q1–Q3)	45 (25–65)	45 (32–63)	0.90	1.0 (0.9–1.0)		
HDL in mg/dL: median (Q1–Q3)	48 (35–64)	47 (38–65.2)	0.66	1.0 (1.0–1.0)		
TG in mg/dL: median (Q1–Q3)	103 (89–163)	106 (92–152)	0.24	1.0 (1.0–1.0)		
WAZ in ZS: median (Q1–Q3)	−1 (−1.9–(−0.1))	−1.2 (−2.4–(−0.6))	0.36	1.1 (1.1–1.2)		
HAZ in ZS: median (Q1–Q3)	−1.3 (−2.1–(−0.5))	−1.5 (−2.5–(−0.6))	0.40	1.1 (1.0–1.1)		
BAZ in ZS: median (Q1–Q3)	−0.8 (−1.9–(−0.1))	−1.2 (−2.2–(−0.1))	0.49	1.2 (1.0–1.5)		
Log_10_pVL: median (Q1–Q3)	4.1 (3.0–5.0)	4.2 (3.3–4.8)	0.93	0.9 (0.6–1.2)		

PDR—pretreatment drug resistance; TB—Tuberculosis, all forms; IPT - isoniazid preventive therapy; CPT—co-trimoxazole preventive therapy; HCT—hematocrit; AST—aspartate transferase; ALT—alanine aminotransferase; Bil-T—total bilirubin; ALB—serum albumin; BUN—blood urea nitrogen; LDL—low density lipoprotein; TG—triglyceride; WAZ—weight-for-age Z score; BAZ—body mass index-for-age Z score; Log10 pVL—baseline viral load (after log transformation); cART—combination antiretroviral therapy; HR—Hazard ratio; aHR—adjusted hazard ratio. ** To avoid collinearity, BAZ (which is a function of weight and height) was excluded from the multivariate model. The following categories were treated as binary variables where “yes” is the reference group—PDR, TB, IPT and CPT. The reference groups for categorical variables are indicated on the table. The following were continuous variables, where hazards were defined in terms of unit decrement—age, AST, ALT, Albumin, BUN, Creatinine, bilirubin and baseline viral load. The following were continuous variables, where hazards were defined in terms of unit increment—CD4 count, hematocrit, total cholesterol, LDL, HDL, TG, WAZ, HAZ and BAZ.
